# Supplementary material for: A fault-intrusion-tolerant system and deadline-aware algorithm for scheduling scientific workflow in the cloud
Source: PeerJ Comput Sci. 2021 Nov 2;7:e747. doi: 10.7717/peerj-cs.747 (PMC8576554; doi:10.7717/peerj-cs.747)
Supplement: Supplemental Information 6 [file peerj-cs-07-747-s006.zip › Myproject1.1/docs/org/cloudbus/cloudsim/class-use/Pe.html]

Uses of Class org.cloudbus.cloudsim.Pe


JavaScript is disabled on your browser.


- Overview
- Package
- Class
- Use
- Tree
- Deprecated
- Index
- Help

- Prev
- Next

- Frames
- No Frames

- All Classes

## Uses of Class org.cloudbus.cloudsim.Pe

- Packages that use Pe

  | Package | Description |
  | --- | --- |
  |  |  |
  | --- | --- |
  | org.cloudbus.cloudsim |  |
  | org.cloudbus.cloudsim.lists |  |
  | org.cloudbus.cloudsim.network.datacenter |  |
  | org.cloudbus.cloudsim.power |  |
- - ### Uses of Pe in org.cloudbus.cloudsim

    Methods in org.cloudbus.cloudsim with type parameters of type Pe

    | Modifier and Type | Method and Description |
    | --- | --- |
    |  |  |
    | --- | --- |
    | `<T extends Pe>  java.util.List<T>` | Host.`getPeList()` Gets the pe list. |
    | `<T extends Pe>  java.util.List<T>` | VmScheduler.`getPeList()` Gets the vm list. |
    | `protected <T extends Pe>  void` | Host.`setPeList(java.util.List<T> peList)` Sets the pe list. |
    | `protected <T extends Pe>  void` | VmScheduler.`setPeList(java.util.List<T> peList)` Sets the vm list. |

    Methods in org.cloudbus.cloudsim that return types with arguments of type Pe

    | Modifier and Type | Method and Description |
    | --- | --- |
    |  |  |
    | --- | --- |
    | `protected java.util.List<Pe>` | VmSchedulerSpaceShared.`getFreePes()` Gets the free pes vector. |
    | `protected java.util.Map<java.lang.String,java.util.List<Pe>>` | VmSchedulerSpaceShared.`getPeAllocationMap()` Gets the pe allocation map. |
    | `java.util.Map<java.lang.String,java.util.List<Pe>>` | VmScheduler.`getPeMap()` Gets the pe map. |
    | `java.util.List<Pe>` | VmScheduler.`getPesAllocatedForVM(Vm vm)` Gets the pes allocated for vm. |

    Method parameters in org.cloudbus.cloudsim with type arguments of type Pe

    | Modifier and Type | Method and Description |
    | --- | --- |
    |  |  |
    | --- | --- |
    | `protected void` | VmSchedulerSpaceShared.`setFreePes(java.util.List<Pe> freePes)` Sets the free pes vector. |
    | `protected void` | VmSchedulerSpaceShared.`setPeAllocationMap(java.util.Map<java.lang.String,java.util.List<Pe>> peAllocationMap)` Sets the pe allocation map. |
    | `protected void` | VmScheduler.`setPeMap(java.util.Map<java.lang.String,java.util.List<Pe>> peMap)` Sets the pe map. |

    Constructor parameters in org.cloudbus.cloudsim with type arguments of type Pe

    Constructor and Description

    `Host(int id,
    RamProvisioner ramProvisioner,
    BwProvisioner bwProvisioner,
    long storage,
    java.util.List<? extends Pe> peList,
    VmScheduler vmScheduler)`

    Instantiates a new host.

    `HostDynamicWorkload(int id,
    RamProvisioner ramProvisioner,
    BwProvisioner bwProvisioner,
    long storage,
    java.util.List<? extends Pe> peList,
    VmScheduler vmScheduler)`

    Instantiates a new host.

    `VmScheduler(java.util.List<? extends Pe> pelist)`

    Creates a new HostAllocationPolicy.

    `VmSchedulerSpaceShared(java.util.List<? extends Pe> pelist)`

    Instantiates a new vm scheduler space shared.

    `VmSchedulerTimeShared(java.util.List<? extends Pe> pelist)`

    Instantiates a new vm scheduler time shared.

    `VmSchedulerTimeSharedOverSubscription(java.util.List<? extends Pe> pelist)`

    Instantiates a new vm scheduler time shared over subscription.
  - ### Uses of Pe in org.cloudbus.cloudsim.lists

    Methods in org.cloudbus.cloudsim.lists with type parameters of type Pe

    | Modifier and Type | Method and Description |
    | --- | --- |
    |  |  |
    | --- | --- |
    | `static <T extends Pe>  Pe` | PeList.`getById(java.util.List<T> peList, int id)` Gets MIPS Rating for a specified Pe ID. |
    | `static <T extends Pe>  Pe` | PeList.`getFreePe(java.util.List<T> peList)` Gets a Pe ID which is FREE. |
    | `static <T extends Pe>  double` | PeList.`getMaxUtilization(java.util.List<T> peList)` Gets the max utilization among by all PEs. |
    | `static <T extends Pe>  double` | PeList.`getMaxUtilizationAmongVmsPes(java.util.List<T> peList, Vm vm)` Gets the max utilization among by all PEs allocated to the VM. |
    | `static <T extends Pe>  int` | PeList.`getMips(java.util.List<T> peList, int id)` Gets MIPS Rating for a specified Pe ID. |
    | `static <T extends Pe>  int` | PeList.`getNumberOfBusyPes(java.util.List<T> peList)` Gets the number of BUSY Pe. |
    | `static <T extends Pe>  int` | PeList.`getNumberOfFreePes(java.util.List<T> peList)` Gets the number of FREE or non-busy Pe. |
    | `static <T extends Pe>  int` | PeList.`getTotalMips(java.util.List<T> peList)` Gets total MIPS Rating for all PEs. |
    | `static <T extends Pe>  boolean` | PeList.`setPeStatus(java.util.List<T> peList, int id, int status)` Sets the Pe status. |
    | `static <T extends Pe>  void` | PeList.`setStatusFailed(java.util.List<T> peList, boolean failed)` Sets the status of PEs of this machine to FAILED. |
    | `static <T extends Pe>  void` | PeList.`setStatusFailed(java.util.List<T> peList, java.lang.String resName, int hostId, boolean failed)` Sets the status of PEs of this machine to FAILED. |

    Methods in org.cloudbus.cloudsim.lists that return Pe

    | Modifier and Type | Method and Description |
    | --- | --- |
    |  |  |
    | --- | --- |
    | `static <T extends Pe>  Pe` | PeList.`getById(java.util.List<T> peList, int id)` Gets MIPS Rating for a specified Pe ID. |
    | `static <T extends Pe>  Pe` | PeList.`getFreePe(java.util.List<T> peList)` Gets a Pe ID which is FREE. |
  - ### Uses of Pe in org.cloudbus.cloudsim.network.datacenter

    Constructor parameters in org.cloudbus.cloudsim.network.datacenter with type arguments of type Pe

    Constructor and Description

    `NetworkHost(int id,
    RamProvisioner ramProvisioner,
    BwProvisioner bwProvisioner,
    long storage,
    java.util.List<? extends Pe> peList,
    VmScheduler vmScheduler)`
  - ### Uses of Pe in org.cloudbus.cloudsim.power

    Constructor parameters in org.cloudbus.cloudsim.power with type arguments of type Pe

    Constructor and Description

    `PowerHost(int id,
    RamProvisioner ramProvisioner,
    BwProvisioner bwProvisioner,
    long storage,
    java.util.List<? extends Pe> peList,
    VmScheduler vmScheduler,
    PowerModel powerModel)`

    Instantiates a new host.

    `PowerHostUtilizationHistory(int id,
    RamProvisioner ramProvisioner,
    BwProvisioner bwProvisioner,
    long storage,
    java.util.List<? extends Pe> peList,
    VmScheduler vmScheduler,
    PowerModel powerModel)`

    Instantiates a new power host utilization history.

- Overview
- Package
- Class
- Use
- Tree
- Deprecated
- Index
- Help

- Prev
- Next

- Frames
- No Frames

- All Classes
